# On Tar Fumigation

**Published:** 1821-11

**Authors:** H. Ward


### *On Tar Fumigation.* By H. Ward.

ANXIOUS to take some part in the performance of the work of rendering service to suffering humanity, I have addressed the present paper to the readers of the Medical Journal; and amply shall I think myself rewarded, should it prove the means of rescuing any individual from the torments of the disease on which it treats. In perusing it, I trust they will bear in mind that the following essay was not written to undergo the severity of criticism; and that, although many parts may be capable of improvement, the subject is one of so much importance to mankind, that the utility of it will make up for what in other respects may be wanting in the style of my composition.

It is deeply to be regretted, that the opinion of the major part of the profession on phthisis pulmonalis, should be so decidedly against the possibility of a cure, that every hint thrown out on that subject is either totally disregarded or looked upon as a matter deserving of no consideration, and from which no possible good can accrue. Some time ago, a pamphlet, written by Dr. Crichton, (a physician of the first eminence in Russia,) on tar fumigation, as a remedy for this disease, was published in London; but I am fearful it shared the same fate with others on the subject. This condemnation does certainly not reflect any great degree of credit on the members of the profession, who have thus slighted a subject of such moment; more especially so, when accompanied with the testimonials of its success from so reputable a source. After the recital of several cases in which it was applied, Dr. Crichton adds, " It must be evident, from the preceding cases, that the tar fumigation, though completely successful in some of them, did not produce the same good effect in all; but, on the other hand, the very great relief which every patient experienced at first from it, particularly in the diminution of cough, expectoration, and hectic fever, is a fact which ought to encourage us to multiply the trials of this remedy as far as possible."* Dr. Crichton used it in a public hospital; but here it did not succeed so well as it otherwise,

---

* Vide *an Account of some Experiments made with the Vapour of boiling Tar, in the Cure of Pulmonary Consumption.* By A. Crichton, M.D. F.R.S. &c. &c.

perhaps, would have done, on account of the numbers which, of necessity, were crammed together in one ward. It would appear, from the Russian writers on this subject, that the most common kind of this disease in that, and I believe in most northern countries, is the scrofulous or tubercular phthisis. It appears that the tar vapour certainly had the property of healing the ulcers in many cases, and also of removing the inflammation of the tubercles. Such being the facts, who will for a moment doubt that the remedy deserves at least a trial. But, in cases of phthisis where, from immense abscesses, disorganization of the lungs has taken place, we must certainly not expect a cure to be performed; though, even in this, very considerable temporary relief may sometimes be afforded.

Shortly after the publication of Dr. Crichton's pamphlet, when speaking to a gentleman, (on the subject of the work,) whose daughter was evidently dying from confirmed pulmonary consumption, he told me, his medical attendant had rejected the idea of using the tar fumigation, alleging it would tend rather to aggravate than remove the disease. Added to a long train of symptoms, her breathing was extremely difficult. She had taken, by the direction of her medical attendant, the foxglove and sulphuric acid in a tonic infusion, and an opiate every night. I advised that the tar fumigation might be used in the manner directed by Dr. Crichton, and was astonished to witness the relief it afforded to the patient, by removing the difficulty of breathing; but, as might be expected, after suffering from her complaint, though certainly in a very mitigated degree, for about twenty days, she died. Another of the cases I witnessed was that of a young man, about twenty-five years of age, whose family were all of a consumptive habit, with the exception of his mother: he was frequently troubled, on taking the least cold, with a most obstinate cough, which generally remained with him for a long time, and, as may be expected, extremely debilitated him. I here also recommended the tar fumigation to him, during the time he was suffering from one of these attacks, and he experienced very considerable relief from its use; and, on any accession of an attack, he can generally cut them short by having recourse to this plan. From this circumstance, I was induced to believe that very great benefit might accrue by a timely use of this remedy in phthisis; and that families who have a predisposition to the disease, (and there are undoubtedly many such,) would, perhaps, be greatly benefitted by the use of it. I have hitherto had no opportunity of trying its use in the first stage of the disease, (except the case of the young man just spoken of,) but entertain so high an opinion of its efficacy, that I should certainly not neglect it in any such cases that came under my care. Of course, such a plan of medical treatment as the more urgent symptoms might point out, would be requisite in conjunction with the remedy in question.

It would, perhaps, be giving this plan of treatment a better trial than could be effected by any other method, to have a building appropriated entirely to the subject; and the wards, instead of being constructed for the accommodation of many patients, should be so formed that one patient only should occupy each; thus preventing any possibility of contagion, even should it be allowed to exist. The philanthropy of some individuals have given rise to similar establishments for the relief of other diseases; and surely no set of patients are more entitled to the assistance of their more affluent brethren than these.

It was on a view of the circumstances contained in the preceding part of this paper, that I was brought to consider the utility this remedy might be of to the asthmatic patient, since the difficulty of breathing in phthisis was so greatly relieved by its use; and, in addition to this, I have always had in view, (although, perhaps, contrary to the opinion of many physicians of the day,) that one of the principal objects in the treatment of asthma is to avoid any disorder of the stomach, or remove it when it was already existing. Now, as the tar fumigation is conveyed directly to the lungs without exciting any stimulus to the digestive organs, such disorder is certainly prevented.

On the first application of this remedy, coughing may, and will in all probability, be produced; mucus will be thrown up, and relief hereby obtained; as the mucus thus expectorated is the peccant matter that causes the asthmatic paroxysm.

The discovery of this fumigation in phthisis did not, however, originate with Dr. Crichton. Some time since, a poor man was in a workhouse in London, incapable of maintaining himself, labouring under a severe cough, which it was feared would terminate in phthisis, in consequence of the hereditary predisposition to that disease which he possessed. In consequence of not being able to follow any laborious calling, he was employed by a wine-merchant in sealing bottles with the resinous composition commonly in use for that purpose. While pursuing this employ, he found his breathing much benefitted; and, not knowing any other source whence this good might arise, he always afterwards, during the more severe attacks of his disorder, would melt the composition in the room where he dwelt, and from this he felt a considerable benefit. Hence, too, as well as from the circumstances before related, was I induced to believe that great advantage might be derived from the fumigation in asthma; I therefore used it in the cases of two patients, who obtained from it a relief far beyond what the most sanguine could expect. In another case it was of very singular benefit. The patient had laborious breathing, strong febrile symptoms, pulse 90, and strong. As venesection and evacuants had been tried in former attacks, without any good effect, they were not now resorted to; but, as dyspœna appeared the most urgent symptom, I had resolved to consider this as the cause of the long train of miseries from which he suffered; and, in consequence, on the next attack, after a mild laxative, I requested the tar fumigation might be used; which was found of great benefit. Since that time, recourse is always had to it on the return of a fit, and I believe with the same success; for I have not seen the patient for a considerable time, in consequence of his residence in a distant part of the country.

I have merely thrown out these hints, with a hope that the attention of the profession will be attracted towards this subject, the importance of which all must be fully aware of; and I sincerely hope it will excite some more able men to investigate it with the attention it deserves.

*Maidenhead, Berks; 26th August,* 1821.

